# Clinical Outcomes and Complications of Cortical Button Distal Biceps Repair: A Systematic Review of the Literature

**DOI:** 10.1155/2016/3498403

**Published:** 2016-07-21

**Authors:** Andreas Panagopoulos, Irini Tatani, Pantelis Tsoumpos, Dimitris Ntourantonis, Konstantinos Pantazis, Ioannis K. Triantafyllopoulos

**Affiliations:** ^1^Department of Shoulder and Elbow Surgery, Patras University Hospital, Papanikolaou 1, 26504 Patras, Greece; ^2^Medical School, University of Athens, Athens, Greece

## Abstract

*Objectives*. The purpose of the present study was to investigate the clinical outcomes and complications of the cortical button distal biceps fixation method.* Material and Methods*. All methods followed the PRISMA guidelines. Included studies had to describe clinical outcomes and complications after acute distal biceps repair with cortical button fixation. Eligibility criteria also included English language, more than 5 cases with minimum follow-up of 6 months, and preferably usage of at least one relevant clinical score (MEPS, ASES, and/or DASH) for final outcome. A loss of at least 30° in motion—flexion, extension, pronation, or supination—and a loss of at least 30% of strength were considered an unsatisfactory result.* Results*. The review identified 7 articles including 105 patients (mean age 43.6 years) with 106 acute distal biceps ruptures. Mean follow-up was 26.3 months. Functional outcome of ROM regarding flexion/extension and pronation/supination was satisfactory in 94 (89.5%) and 86 (82%) patients in respect. Averaged flexion and supination strength had been reported in 6/7 studies (97 patients) and were satisfactory in 82.4% of them. The most common complication was transient nerve palsy (14.2%). The overall reoperation rate was 4.8% (5/105 cases).* Conclusion*. Cortical button fixation for acute distal biceps repair is a reproducible operation with good clinical results. Most of the complications can be avoided with appropriate surgical technique.

## 1. Introduction

*Rationale.* Distal biceps tendon ruptures are estimated to occur at a rate of 1.2 per 100,000 persons per year and are most commonly seen in the dominant elbow of men who are in the fourth decade of life [1]. A single traumatic event in which an unexpected eccentric force is applied to a flexed elbow is the most common mechanism of injury. Tobacco and anabolic steroid use and the use of statin drugs are known to be associated with an increased risk of distal biceps tendon ruptures [1, 2].

Results of surgical repair have been superior to nonsurgical treatment in terms of improving elbow strength in flexion and supination, as well as overall upper extremity endurance [3, 4]. Single-incision techniques and two-incision approaches have been described using a variety of fixation methods, including transosseous suture repair [5–7], suture anchors, [8, 9] cortical button fixation, [7, 10–13] double intramedullary cortical button, [14] interference screws (alone [15] or in conjunction with a cortical button [16]), and endoscopic assisted techniques [17].

Cortical button repair of distal biceps tendon ruptures was first described by Bain et al. in 2000 [10]. Biomechanical studies have demonstrated higher load to failure when compared to other techniques [18, 19] thus allowing for earlier postoperative rehabilitation [20]. Excellent clinical results of the cortical button technique have been reported with respect to patient satisfaction and restoration of functional outcome with minimal complications [10, 11, 21]. Recently, Chavan et al. [22] in a systematic review showed that repairs using a cortical button performed better than other repair methods. The authors also compared two different approaches and found no difference in the overall incidence of complications between 2-incision approaches (16%) and single-incision approaches (18%), although they noted more instances of loss of forearm rotation with the 2-incision approach.

Clinical studies that report on clinical outcomes and complication rates of cortical button fixation are scarce and are generally of small numbers with low levels of evidence [7, 23–28]. Despite the reported good clinical results and high patient satisfaction, the technique has been associated with several complications such as heterotopic ossification (HO), nerve injuries, and failure of the repair [25, 29–33].

*Objectives.* To our knowledge, a systematic review of clinical outcomes and complications after cortical button fixation for acute distal biceps ruptures has not been performed yet. The purpose of the present study is to critically evaluate the relevant literature to better quantify the expected outcomes and complications in a larger patient population. Such information would be potentially helpful in developing an evidence-based approach in the management of these injuries.

## 2. Material and Methods

### 2.1. Identification of Studies

**Figure 1:**
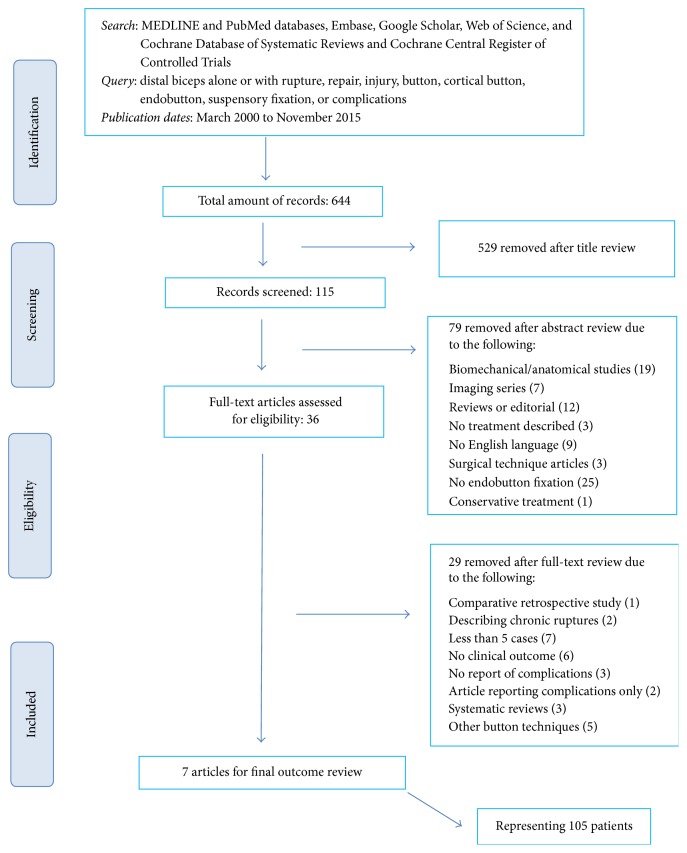
Search methodology and flowchart of excluded studies.

A research protocol was developed as described by Wright et al. [34] and used throughout the study process. This protocol was not registered. All methods followed the PRISMA guidelines. Analytic searches of PubMed, Embase, Web of Science, Google Scholar, and the Cochrane Database of Systematic Reviews and Cochrane Central Register of Controlled Trials were performed, restricting search results to the years 2000, when the technique was first reported, through May 2015. The query was distal biceps alone or with rupture, repair, injury, button, cortical button, endobutton, suspensory fixation, and/or complications (Figure 1). All titles and abstracts were reviewed to identify potentially relevant articles. The full manuscript was retrieved for all potentially relevant articles and when the title, keywords, or abstract revealed insufficient information to determine appropriateness for inclusion. The bibliographies of the retrieved studies were manually checked for potential relevant articles that were missed in the initial search. Second-stage screening of the full-text articles was performed unblended by 2 of the authors (A. Panagopoulos and I. Tatani). Duplicates were deleted. On November 30, 2015, we updated the search to provide a complete up-to-date interpretation of available data. Disagreements were discussed and resolved in consensus.

### 2.2. Eligibility Criteria

Clinical trials, observational studies, and case series involving patients with distal biceps ruptures treated with cortical button fixation from 2000 onwards were included. To be eligible regarding the final outcome, studies had to describe at least 1 of the following functional outcome measures: (1) range of motion (ROM) (flexion/extension, pronation/supination); (2) strength of the elbow after and before surgical treatment or strength of the elbow after surgical treatment compared with the contralateral side (unaffected elbow); (3) at least one relevant score (MEPS, ASES, or DASH); and (4) complication type and rates. Eligibility criteria also included English language, acute repairs (<6 weeks after injury), more than 5 cases, and minimum follow-up of 6 months. We excluded studies of other distal biceps fixation methods (suture anchors, transosseous sutures, double button fixation, or cortical button with supplementary interference screw) as well as studies conducted on children (mean age < 18), cadavers, review and editorial articles, or anatomical and biomechanical studies.

## 3. Data Extraction

Included studies were divided into 2 groups based on patient's demographic and clinical data.* Group A* included studies presenting comprehensive patient flowcharts with complete demographic, outcome, and complications data, thus allowing us to extract separate information for the acute cases.* Group B* included studies that met our inclusion criteria but presented their data in mean values without separate information for each patient. Unlike similar reviews, in the present study, we decided to exclude chronic ruptures (older than 6 weeks) supposing that the overall clinical outcome and complication rate would be worse in comparison with acute repairs. In accordance with Chavan et al. [22], the functional outcome of ROM and strength was divided into satisfactory or unsatisfactory. A loss of at least 30° in motion (flexion, extension, pronation, or supination) and a loss of at least 30% of strength were considered an unsatisfactory result. A loss of <30° in motion and a loss of <30% of flexion or supination strength were considered a satisfactory result. Heterotopic ossification was not considered a complication unless it was noted to be associated with pain or to cause a loss of greater than 30° of motion in any plane or required revision operation. All inclusion and exclusion criteria as well as our definitions of complications were defined before performing the literature review. Each clinical study was given a level of evidence by consensus agreement of the investigators [35]. The heterogeneity and low level of evidence of the studies that met our inclusion criteria prevented us from performing a meta-analysis.

## 4. Results

The final trial selection identified a total of 644 study records. After screening of the titles, the literature search yielded 115 studies that were eligible for abstract assessment. After screening of the abstracts and removal of duplicates, the literature search yielded 36 studies that were eligible for full-text assessment. Seven of the reviewed articles met our inclusion and exclusion criteria. Two of the included articles reported on 2 study groups; one study compared two different protocols of rehabilitation [20] and one study compared patients with or without complications [20, 23]. Three studies referred to acute repairs only [20, 23, 27] and four studies presented a mixed population predominantly of acute repairs [10, 11, 21, 26]. The included studies reported on 126 patients with 127 acute or chronic distal biceps ruptures. After removal of chronic cases (16), partial ruptures (4), and revisions of acute repairs (1), the final study group represented 105 patients that were included in our systematic review.

All clinical studies were designated level IV evidence by both reviewers; there were no randomized, prospective, or retrospective comparative studies. According to our criteria, 4 studies were included in Group A (having patient flowchart), thus allowing us to extract personalized data for each patient with acute repair, and 3 studies were included in Group B where data were extracted as means and percentages. All eligible studies were case series composed of minimum 5 to maximum 27 patients per study (Table 1). The heterogeneity and level of evidence of the studies that met the inclusion criteria prevented us from performing a meta-analysis. A single anterior approach was used in all patients.

### 4.1. Clinical Outcome (Table 2)

The sex distribution was 98% male (103 patients) and 2% female (2 patients). The average age of the patients at the time of injury was 43.6 years. Mean follow-up time was 26.3 months (range from 6 to 60 months). All studies reported elbow range of motion (flexion/extension, pronation/supination) at the latest follow-up. According to our criteria, the functional outcome of ROM regarding flexion/extension and pronation/supination was satisfactory in 94 (89.5%) and 86 (82%) patients, respectively. There was a ROM deficit of >30° in flexion/extension in 11 patients (10.5%) and a ROM deficit of >30° in pronation/supination in 18 patients (18%). Strength was reported for 92.3% of the patients in the included studies. Flexion and/or supination strength was diminished by >30% compared with the contralateral elbow in 17 of 97 patients (17.5%). A clinical performance score was utilized in five studies at the latest follow-up: 1 study used the DASH score only, 1 the ASES score, 1 the MEPS score, 1 the MEPS and DASH score, and 1 study the DASH, MEPS, and ASES scores. One study reported better DASH score in patients that have not received any postoperative physiotherapy [20] and one study reported significant lower scores of MEPS, ASES, and DASH in patients having complications (9 cases) after biceps repair in comparison to patients having no complications (18 cases) [23].

### 4.2. Complications (Table 3)

Complications were documented in all included studies and are presented in Table 3. There were overall 29 complications (27.6%), of which 15 were neurologic disorders (14.2%). Neurapraxia of the lateral antebrachial cutaneous nerve (LABCN) was the most common complication, accounting for 9 cases (8.6%). There were also four transient posterior interosseous nerve (PIN) palsies (3.8%) and two persistent superficial radial nerve (SRN) palsies (1.3%). Wrong button placement or disengagement and wound problems were the second-most common complications accounting for 3.8% each. Heterotopic ossification (HO) was found in 2 studies. In the first [21], there were 2 asymptomatic cases not included in the final incidence of HO and in the second [23] there were 3 symptomatic cases of which one had been operated twice. The overall incidence of HO in this review was 3/105 cases (2.9%). Other complications included drainage of abscess in one case and 2 reruptures of the reconstructed tendon. The overall reoperation rate was 4.8% (5 of 105 cases).

## 5. Discussion

The clinical studies on outcomes of suspensory cortical button fixation for distal biceps ruptures are few, based on retrospective study designs, and often unclearly reported. We are unaware of any previously published systematic review of a similar nature. Watson et al. [36] recently performed a systematic review of various repair techniques for acute distal biceps tendon ruptures. Twenty-two studies met authors' inclusion criteria with a total of 494 patients; the authors reported an overall complication rate of 26.4% for suture anchors, 20.4% for bone tunnels, 44.8% for intraosseous screws, and 0% for cortical button fixation. In their report, however, only 3 of 22 studies used cortical button fixation in 17/494 (3.5%) patients, thus resulting in a sample size that may have been inadequate for comparing complication rates between this and other fixation techniques. More recently, Kodde et al. [37] performed a systematic review of clinical outcome and complication rates in 1074 patients divided into 4 fixation groups: suture anchors (565 patients), bone tunnels (321 patients), interference screws (42 patients), and cortical buttons (147 patients, 13.6%). They found no significant difference in range of motion and strength between the different approaches and fixation techniques. The double-incision approach had significantly fewer complications than the single-incision anterior approach, and the bone tunnel fixation had significantly fewer complications than the other 3 fixation techniques. However, as the double-incision approach was used with bone tunnel fixation in 84% of cases, there was a strong interrelationship between these variables. With the present systematic review, we were able to extract data for 105 patients treated acutely (within 6 weeks) with cortical button fixation and to analyze clinical outcome and complication rates in a larger homogenized patient population. However, a considerable risk of biases can be attributed to several important factors such as the surgical approach, the rehabilitation protocol, the mixed population of presented cases without universal outcome scoring, the optimal surgical technique, and the lack of long-term radiological evaluation that could underestimate the incidence of heterotopic ossification; these factors will be discussed further.

### 5.1. Surgical Approach

The single anterior operative incision was used in all cases. It should be noted that previews reviews have shown a higher complication rate with the two-incision approach. In their systemic review, Watson et al. [36] found that two-incision techniques can replicate the biceps footprint more anatomically, with equal or lower complication rates when compared with one-incision techniques. However, as the number of patients who underwent two-incision repair was small, the studies were too underpowered to permit an accurate comparison between the two groups. El-Hawary et al. [38] compared one-incision repair with a modified two-incision repair and found significantly greater elbow flexion in the one-incision group. Isokinetic and isometric elbow flexion strength were also significantly greater in the one-incision group in early follow-up, but the two groups equalized at one year. Chavan et al. [22] in a recent systematic review reported no difference in overall incidence of complications between 2-incision approaches (16%) and single-incision approaches (18%), but there were significantly more instances of loss of forearm rotation with the 2-incision approach. Johnson et al. [39] performed a retrospective comparative study in 26 patients (12 cases with single incision with suture anchors and 14 cases with two incisions through bony tunnels) and found no statistically significant differences in regard to flexion strength or endurance, supination strength or endurance, or complication rates between the two techniques. Finally, Kodde et al. [37] found significantly fewer complications using the double-incision approach compared with the single-incision anterior approach and also fewer complications comparing the bone tunnel fixation technique with the other fixation techniques. However, in addition to the differences in approach between the groups, there was also a difference in fixation technique. This raises the question of whether the differences found between the groups were caused by the approach or the fixation technique or a combination of both and the authors were unfortunately not able to efficiently disentangle the effects of these variables. In the present review, the use of the same anterior approach in all patients may represent one of its major strengths, as it allows analysis of a homogenous population treated with the same surgical approach and fixation technique.

### 5.2. Rehabilitation Protocol

Recent anatomical and biomechanical studies [18, 19, 40] have demonstrated higher load to failure of cortical button when compared to other fixation techniques thus allowing for immediate postoperative rehabilitation. Rose et al. [41] showed that unrestricted early active motion with a 0.9 kg weight restriction may be possible immediately after the repair. In the present review, there is no consensus regarding the postoperative rehabilitation protocol. It was interesting that only 1 study suggested prompt elbow range of motion after the repair [21]. Most authors utilized postoperative application of long arm cast or splint in 90 degrees of flexion for one or two weeks and active ROM thereafter. Heavy lifting is not allowed for 2-3 months. Two studies limited extension to 30–40 degrees for 3–6 weeks. One study reported use of long arm cast for 6 weeks. From the present data, we could not perform a quantitative data synthesis for the optimal physiotherapy protocol. Despite the proven ultimate strength of cortical button fixation, it seems that most surgeons prefer a short period of immobilization in a cast or splint and gradual nonrestricted ROM thereafter. The study of Spencer Jr. et al. [20] compared supervised physiotherapy (6 patients) against unrestricted ROM (9 patients) and found significant difference for time to full ROM (8.67 weeks for the supervised therapy group and 4.38 weeks for the unrestricted group). There were no significant differences in final ROM or DASH scores. These data suggest that unrestricted ROM can result in a quicker return to full ROM without an increased risk of rerupture and are recently supported from Smith and Amirfeyz's study [42] who suggested that immediate mobilization after distal biceps tendon repair using a single anterior incision and a cortical button system seems to be safe, with no tendon rerupture and excellent clinical outcomes.

### 5.3. Mixed Data and Lack of Universal Outcome Scoring

Unlike similar reviews, in the present study, we decided to exclude chronic ruptures (older than 6 weeks) supposing that the overall clinical outcome and complication rate would be worse in comparison with acute repairs. Also, several studies were excluded during the eligibility process because they did not have separate analytic data and differentiation between acute and chronic cases. Dillon et al. [26], however, compared the clinical results between acute (17) and chronic (10) ruptures, finding no significant difference in flexion strength, flexion endurance, supination strength, or supination endurance between the two groups. The clinical outcomes (ASES) were also similar between the two groups. These chronic cases (10 patients) were excluded from our review. Anakwenze et al. [43] performed a retrospective comparative study between acute (12) and chronic (6) ruptures and found similar clinical and radiological outcome without any complications at one-year follow-up. This low complication rate in chronic repairs contrasts with the findings of other reviews, such as Cain et al. [25] who reported on a total of 198 patients; the authors found a 46% complication rate in patients operated upon for greater than 4 weeks from injury compared to a rate of 30% in those operated upon acutely. With the exception of objective elbow ROM (flexion/extension, supination/pronation) which had been recorded in all studies, the other instruments used in the original articles to determine functional outcomes were not homogeneous. As a result, statistical analysis to identify significant differences associated with outcome scores was not performed. In future studies design, we suggest not only the utilization of objective ROM and isokinetic flexion and supination strength but also the application of at least one performance elbow score (MEPS, ASES, or DASH) and one score for general health status (SF-12).

### 5.4. Comparison of Complications

Complications after repair of distal biceps can be divided into major complications such as posterior interosseous nerve (PIN) palsy, rerupture, reoperation, and symptomatic heterotopic ossification and minor complications such as temporary paresthesia (lateral antebrachial cutaneous nerve (LABCN), superficial radial nerve (SRN)), superficial infection, problems with wound healing, and irritation from the cortical button.

#### 5.4.1. Nerve Injury

In the present review, there were 4 transient PIN palsies (3.8%), 9 LABCN palsies (8.6%), and 2 SRN palsies (1.9%). One study [23] included 53% (8/15) of these injuries and all the cases of transient PIN palsies. PIN palsy is a relatively rare but serious complication after distal biceps repair using cortical button fixation [32]. Possible mechanisms of injury include the wrong trajectory of the guide pin [44, 45], irritation from the button [46], or excessive compression of the nerve from the retractors during radial tuberosity preparation. Nigro et al. [33] performed a retrospective review using electronic records of patients who underwent distal biceps repair via one-incision anterior approach and found an incidence of transient PIN palsy of 3.2% (9 of 180 patients) which is in accordance with the results of the present review. The fixation method was suture anchors in 3 patients and cortical button in six. Cain et al. [25] reviewed complication rates in 198 patients with distal biceps rupture (119 acute and 79 chronic) treated with three different fixation methods (anchors, bone tunnels, and cortical button) via a single anterior approach in 93% of the patients. The incidence of PIN palsy in the 69 patients treated with suspensory button fixation was 6%, whereas for LABCN and SRN palsies it was 30% and 3%, respectively. These figures are significantly higher than in our report, which according to the authors could be attributed to the high percentage of late reconstructions in their study. LABCN paresthesia resolved spontaneously in all but 2 patients in our review, again in the same complication study [23] that had also the only cases of persistent RSN paresthesia. Smith and Amirfeyz [42] reported an unusual high rate of transient nerve paresthesia concerning almost two-thirds of their patients. This involved the superficial radial nerve in 6 patients (27%) and the lateral antebrachial cutaneous nerve in 8 patients (36%). The authors suggested that their measurement technique using a 0-to-10 analog scale compared with the uninjured arm proved to be a very sensitive tool in detecting any sensory deficit and perhaps explains the high incidence. This complication may be underreported as many patients with mild sensory deficits remain unaware unless they received proper clinical examination, and therefore the complication is underreported.

#### 5.4.2. Heterotopic Ossification

HO is a well-known complication of distal biceps repair. Many mechanisms have been postulated as causative factors such as the bone debris at surgery, hematoma formation, and muscle injury either from the initial injury or from surgical dissection [47]. Single-incision approaches, minimum muscle trauma during surgery, and thorough fluid irrigation of the surgical bed to remove bony debris have been proposed to reduce the risk of symptomatic HO. In the present review, the overall incidence of HO was 2.9%. Only 3/105 of the included patients were symptomatic with one operated on twice for ectopic bone removal. In the literature, there are only sporadic case reports of symptomatic HO after distal biceps repair with cortical button fixation [29, 31, 34]. Agrawal and Stinson [29] performed surgical excision of the exostosis at approximately 5 months postoperatively due to 20-degree motion loss in supination. They found no compromise of the tendon repair. Preoperatively the patient received irradiation and postoperatively a 6-week course of indomethacin. Dillon and Lepore [31] treated their patient conservatively as he refused surgical intervention and they reported satisfactory outcome at 14-month follow-up. Vidal et al. [48] found unexpected high incidence of HO (50%) in 4 of 8 patients treated with single-incision suspensory button fixation. In three patients, the ectopic bone was excised and the final functional outcome was good. In contrast, Cain et al. [25] found a low (3%) incidence of HO in patients treated with cortical button fixation. The same percentage was found also in 119 patients treated with sutures anchors. Finally, Kodde et al. [37] in their recent systematic review reported 19/146 (13%) cases with HO after cortical button distal biceps repair, but this was severe in only two patients (1.3%). In the present review, routine use of radiographic control was not performed by all authors in these series and the incidence of HO may have been underestimated.

#### 5.4.3. Rerupture

Rerupture of the reconstructed biceps is a relatively uncommon complication [25, 30]. In the present review, we found 2 out of 105 (1.9%) cases of complete rerupture of the reconstructed tendon. Cain et al. [25] reported no tendon reruptures in 69 cortical button repairs compared to 4 reruptures in 119 patients using suture anchor fixation whereas Kodde et al. [37] in their systematic review reported only 2 cases in 146 cortical button fixations (1.3%).

### 5.5. Optimal Surgical Technique

It is important to discuss in detail one of the articles (Banerjee et al. [23]) which showed an unusual high rate of complications after cortical button biceps repair in only acute cases (27 patients). This report actually includes all the PIN palsies, all of the LABCN that did not recover, all of the SRN palsies, the only case of HO that required surgery, one of the 2 cases of disengagement of the button, all wound healing problems, one of the two reruptures, and 60% of all the revision surgeries. Overall, this report had 16 complications in 27 patients (59%) in comparison with 13 complications in the remaining 78 patients (16.6%). The authors have noticed in their discussion this high rate of complications which could be attributed to several factors such as the use of Hohmann retractors at both sides of the radius, the trajectory of the drilling (more radially and distally), the length of skin incision, and the number of different surgeons, both experienced and not, performing the operations. Due to this high rate of complications, the authors have changed their approach to button biceps repair; senior surgeons perform now the operation; they utilize more vertical pin trajectory with the elbow in extension and full supination, smaller incision, and use of skin hooks instead of retractors. These surgical tips are important for optimal outcome and avoidance of serious complications. Furthermore, intraoperative fluoroscopy and thorough irrigation of the wound after drilling with prophylactic radiation and/or indomethacin administration have also been suggested for correct button placement and avoidance of HO [10, 11, 26, 28], respectively.

### 5.6. Strengths and Weaknesses

The designs of the included studies in this systematic review did not allow for quantitative data synthesis. However, this study is not without its strengths. We were able to perform a thorough review of the current literature on acute distal biceps ruptures using cortical button fixation with respect to range of motion, flexion and supination strength, complications after surgery, and clinical outcomes. We used strictly defined, prospective criteria for inclusion and exclusion of patients and definition of complications, and we were able to extract patient data for 105 patients with acute repairs. With the exception of one study [23], we were able to demonstrate not only good clinical outcomes but also a low incidence of serious complications such as PIN palsy heterotopic ossification reruptures and reoperations. Another important finding of our study is that full restoration of ROM and strength, especially pronation/supination, may not be finally obtained as almost 18% of the patients in this review showed an unsatisfactory result. It should be also noted that clinical superiority of cortical button fixation in contrast to other fixation techniques has not yet been confirmed in the literature. Recordon et al. [7] reported recently (2015) a retrospective comparative study between cortical button (19 patients) and transosseous suture fixation (27 patients) in acute distal biceps ruptures utilizing a 2-incision approach and found no significant statistical differences in subjective patient evaluation, pain, range of motion, supination strength, and overall complications. Despite prompt immobilization in a cast for 6 weeks in the transosseous group, the clinical performance was similar at the latest follow-up. Further well-designed, prospective, comparative studies are needed to prove the superiority, if any, of cortical button distal biceps repair.

## 6. Conclusions

Our study shows that a single-incision endobutton repair of acute distal biceps ruptures is a reproducible operation with good clinical results and relatively low incidence of severe complications that can be avoided with attention to appropriate surgical technique. Patients have to be informed that they may have ROM and strength deficits, especially in pronation/supination, and incur a significant risk for transient sensory nerve neurapraxias.

## Figures and Tables

**Table 1:** Demographic data, included patients, surgical approach, and physiotherapy protocol.

First author	Patients/cases	Acute/chronic^#^/ partial^%^/revisions^@^	Included patients	Mean age (y) (range)	Mean follow-up [m] (range)	Surgical approach	Comments	Postoperative physiotherapy protocol
(1) Bain, 2000 [10]	12	10/2^#^	10	39 (25–50)	16 (8–29)	SA		Cast for 1 week, motion as tolerated, no heavy lifting for 3 months
(2) **Greenberg, 2003** [11]	14	11/3^#^	11	45 (NR)	20 (13–28)	SA		Bulky dressings, after 3-4 days' full ROM, no extension > 30°, full use in 3 months
(3) **Spencer Jr.,** ** 2008 **[20]	15	15	15	46 (31–64)	23 (12–36)	SA	Group 1^*∗*^ (6 pt) Group 2^$^ (9 pt)	Both groups cast for 2 weeks (Group 1, hinged brace for another 4 weeks), extension limited to 40°, full extension in 6 weeks
(4) Peeters, 2009 [21]	23	17/2^#^/4^%^	17	50 (39–58)	16.8 (6–48)	SA		Immediate full ROM
(5) Dillon, 2010 [26]	27	17/9^#^/1^@^	17	50.1 (41–62)	30.9 (15–50)	SA		Cast for 2 weeks and then active ROM, in 4 weeks' ADL, no lifting > 5 lbs
(6) Gupta, 2012 [27]	8/9	9	8	27.4 (21–42)	41.5 (24–60)	SA		Removable splint for 2 weeks with active ROM, ADL, and no heavy lifting for 6 weeks
(7) **Banerjee, 2013 **[23]	27	27	27	47.9 (34–63)	36.1 (NR)	SA	Group 1^!^ (19 pt) Group 2^&^ (9 pt)	Cast for 6 weeks, passive ROM started after 3rd week

*Total*	*126/127*	*106/16/4/1*	*105*	*43.6*	*26.3*			

^#^More than 6 weeks after injury; ^%^partial repairs; ^@^revision repairs; SA: single anterior approach; NR: not reported; ROM: range of motion; ADL: activities of daily living; Group 1^*∗*^: supervised physiotherapy; Group 2^$^: no therapy; Group 1^!^: without complications; Group 2^&^: with complications; studies in Group B are highlighted with bold.

**Table 2:** Clinical results of included studies.

Main author	Number of patients	Range of motion							Strength testing				Mean MEPS	Mean ASES	Mean DASH
		Flexion/extension			Supination/pronation				AVFS (%)	AVSS (%)	SAT	NS			
		AVROM	SAT	NS	AVP	AVS	SAT	NS							
**Bain [10]**	10	5°–146°	9	1	80	81	9	1	NR	NR	10	—	NR	NR	NR

**Greenberg **[11]	11	−4°–141°	11	—	73	74	11	—	97	82	11	—	NR	NR	NR

**Spencer Jr. **[20]	15								100	100	15	—	NR	NR	
**Group **1^*∗*^ ** (6 pt)**		−1.5°–139.1°	6	—	75.8	76.6	6	—							3.1
**Group **2^$^ ** (9 pt)**		−1.5°–138.5°	9	—	77.5	76.5	9	—							1

**Peeters **[21]	17	−2°–134°	16	1	77	76	12	5	80	91	11	6	93.8	NR	NR

**Dillon **[26]	17	0°–135°	17	—	67	72	13	4	101	99	15	2	NR	98.7	NR

**Gupta **[27]	8	0°–143°	8	—	77	81	8	—	NR	NR	—	—	100	NR	0

**Banerjee **[23]	27														
**Group **1^!^ ** (18 pt)**		1.1°–147.2°	18		89.4	85.8	18		94	92.9	18		100	98	0.3
**Group **2^&^ ** (9 pt)**		−1.7°–142.2°		9	88.9	78.3		9	88	77.6		9	77.8	87.8	5.2

*Total *	*105*		*94 (89.5)%*	*11 (10.5%)*			*86 (82%)*	*19 (18%)*			*80 (82.4%)*	*17 (17.6%)*			

Group 1^*∗*^: supervised physiotherapy group; Group 2^$^: no therapy; Group 1^!^: without complications; Group 2^&^: with complications; MEPS: Mayo Elbow Performance Score; ASES: American Shoulder & Elbow Surgeons Score; DASH: Disabilities of the Arm, Shoulder & Hand Score; NR: not reported; AVROM: average range of motion; AVP: average pronation; AVS: average supination; AVFS: average flexion strength; AVSS: average supination strength; SAT: satisfied; NS: nonsatisfied; studies in Group B are highlighted with bold.

**Table 3:** Complications of distal biceps cortical button fixation.

Authors	Patients	PIN palsy	LABC palsy	SRN palsy	Infection	Heterotopic ossification	Cortical button problems	Wound problems	Rerupture	Re-op
(1) Bain et al. [10]	10	—	—	—	1 abscess 6 m postop (drainage)	—	—		—	1
(2)** Greenberg et al. **[11]	11	—	3 (transient)	—	—	—	—	1 (wound irritation, resolved spontaneously)	—	—
(3)** Spencer Jr. et al. **[20]	15	—	2 (transient)	—	—	—	—		—	—
(4) Peeters et al. [21]	17	—	—	—	—	2 (asymptomatic)^*∗*^	3 (2 wrong placement cases, 1 disengagement case)		1	1
(5) Dillon et al. [26]	17	—	2 (transient)	—	—	—	—		—	—
(6) Gupta et al. [27]	8 (1 bilateral case)	—	—	—	—	—	—		—	—
(7)** Banerjee et al.** [23]	27	4 (transient)	2 (not resolved)	2 (persistent)	—	3 (1 severe case, removed twice)	1 disengagement case	3 (wound healing disorder)	1	3

*Total*	*105*	*4 (3.8%)*	*9 (8.6%)*	*2 (1.9%)*	*1 (0.9%)*	*3 (2.9%)*	*4 (3.8%)*	*4 (3.8%)*	*2 (1.9%)*	*5 (4.8%)*

PIN: posterior interosseous nerve; LABC: lateral antebrachial cutaneous nerve; SRN: superficial radial nerve; Re-op: reoperation studies in Group B are highlighted with bold letters; ^*∗*^not included as complication.
